# In vitro study of the modulatory effects of heat-killed bacterial biomass on aquaculture bacterioplankton communities

**DOI:** 10.1038/s41598-022-23439-8

**Published:** 2022-11-16

**Authors:** J. M. G. Sousa, A. Louvado, F. J. R. C. Coelho, V. Oliveira, H. Oliveira, D. F. R. Cleary, N. C. M. Gomes

**Affiliations:** CESAM-Centre for Environmental and Marine Studies, Department of Biology, University of Aveiro, 3810-193 Aveiro, Portugal

**Keywords:** Applied microbiology, Microbiology techniques, Microbial ecology

## Abstract

Recent studies have shown that the addition of non-viable microbial biomass or their components (postbiotics) to fish feed can modulate the gut microbiome and positively influence fish health in aquaculture systems. However, no information was hitherto available on the use of non-viable microbial biomass to manipulate aquaculture bacterioplankton communities. To fill this gap, here we used an in vitro model to assess the effects of heat-killed biomasses of an antagonistic strain *Pseudoalteromonas rubra* SubTr2 and a non-antagonist strain *Escherichia coli* DH5α on bacterioplankton communities of a recirculating aquaculture system (RAS). Our results showed that these biomasses can have generalist and species-specific effects on aquaculture bacterioplankton structure and function. In addition, they enriched the abundance of bacterial predators, reduced bacterial load and potentially influenced nutrient cycling and pathogen development in aquaculture water. Despite its preliminary nature, for the first time, this study showed that heat-killed microbial biomass has potential application as an in situ modulator of bacterioplankton in aquaculture systems.

## Introduction

Despite the well-known benefits of using probiotics in aquaculture^1,2^, previous studies have shown that their health-promoting effects can be independent of their viability^3^. In consequence, non-viable probiotics, known as postbiotics, have been developed. These have been recently defined, by the International Scientific Association of Probiotics and Prebiotics (ISAPP), as “inanimate microorganisms and/or their components capable of conferring observable health benefits to their hosts”^4^. The exact mechanism of this beneficial effect is, however, still not fully understood. Various studies have suggested that bacterial cell components contribute through an induced immunomodulatory reaction of the host, an interaction with the host’s gut microbiota or a protective effect due to hypocholesterolemic, hypolipidemic anti-inflammatory, anti-tumour and/or antioxidant properties of the postbiotic compounds (Aguilar-Toalá et al.^3^ and citations therein). Furthermore, the use of non-viable microorganisms has been suggested as a means of avoiding the environmental concerns and logistical constraints associated with live bacteria^2,4^.

Unlike probiotics, postbiotics are incapable of colonizing the fish gut, but evidence suggests that they are capable of modulating the structure and function of the gut microbiome, leading to improved immunity and growth performance^2^. This ability of non-viable microorganisms to promote biological responses, similar to those of probiotics, has already been demonstrated using in vivo fish models. For example, feed supplementation with heat-killed microbial biomass of lactic acid bacteria has been shown to improve growth, feed efficiency, immunology and survival rate in animal hosts^5–11^. Supplementation with non-viable *Bacillus amyloliquefaciens*; *B. clausii*, *Pseudomonas aeruginosa*, *Gordonia bronchialis* and *Pediococcus pentosaceus* was associated with similar improvements^7,12–16^. In addition to this, feed supplementation with non-viable *Lactococcus garvieae*, *Rhodotorula minuta* and *Cetobacterium somerae* significantly modulated gut bacterial community structure and function^17,18^.

In the present study, we discuss the potential application of non-viable biomasses to manipulate the aquaculture bacterioplankton in recirculating aquaculture systems (RAS). Since all aquaculture compartments (e.g., water, biofilters and fish skin and gut) are in direct contact with planktonic microbes, bacterioplankton will contribute to the dispersion of both beneficial and pathogenic bacteria and play a critical role in shaping the aquaculture microbiome^19,20^. Therefore, bacterioplankton community manipulation may have consequences for the whole aquaculture system. In line with the modulatory effects of non-viable biomasses, in this study we aimed to use an in vitro model to assess the effects of heat-killed biomasses of an antagonistic strain *Pseudoalteromonas rubra* SubTr2 and non-antagonistic strain *Escherichia coli* DH5α on RAS bacterioplankton structure and putative function. While *E. coli* DH5α is a non-antibiotic producer strain widely used in molecular studies and in a variety of antimicrobial test methods, *P. rubra* strains are known to produce pigments with biotechnological potential, including antimicrobial activities^21–23^.

*Pseudoalteromonas* is a genus consisting of aerobic marine bacteria known to inhibit the growth of various putative pathogenic bacteria (e.g., *Vibrio harveyi*, *Vibro nigripulchritudo*, *Vibrio anguillarum* and *Vibrio parahaemolyticus*)^22,24^ and microeukaryotes, including algae and fungi^25,26^. Various strains of *P. rubra* have been shown to inhibit growth of the aquaculture pathogen *V. anguillarum*^24^. Due to their antagonistic properties, a great interest has emerged in using *Pseudoalteromonas* species for pathogen suppression in aquaculture systems^21,22^. Its use as a probiotic supplement in feed has been evaluated, in vitro and in vivo*,* across a diverse range of marine organisms (e.g., abalone *Haliotis tuberculate*^21^, turbot *Scophtalmus maximus*^27^, and prawn *Litopenaeus stylirostris*^28^). In recent studies, the addition of living cells of *Pseudoalteromonas* strains hCg-6 and NC201 to rearing water (10^5^–10^6^ CFU mL^−1^) was shown to significantly increase host survival (abalone and prawns, respectively) in an infection-inducing challenge experiment with pathogenic strains of *V. harveyi* and *V. nigripulchritudo*, respectively^21,28^. In addition to this, *Pseudoalteromonas* NC201 reduced the prevalence of *V. nigripulchritudo* in prawns. However, to the best of our knowledge, no study has yet investigated the modulatory effects of heat-killed biomasses of *Pseudoalteromonas* on aquaculture bacterioplankton communities.

## Materials and methods

### Bacterial strains

The bacterial strain SubTr2 was previously isolated from marine sponges during the Ecotech-Sponge project (PTDC/BIA‐MIC/6473/2014–POCI‐01‐0145‐FEDER‐016531). Briefly, sponge samples were aseptically cut into small pieces and homogenized with autoclaved sterile natural seawater (NSW) using a mortar and pestle. Each homogenate was serially diluted in NSW and then spread onto half-strength marine broth agar supplemented with selective agent chloramphenicol (30 µgml^-1^) or trimethoprim (5 µgml^-1^) or mercury (II) chloride (20 µgml^-1^) or sodium (meta) arsenite (15 µgml^-1^). Plates were incubated at 17 °C for up to 7 days. Bacterial growth was monitored daily, and distinct colony morphotypes were selected for isolation. Pure cultures were obtained and stocked in marine broth containing 20% (v/v) glycerol at − 80 °C.

The DNA of this strain was extracted using FastDNA SPIN Kit for Soil (MP Biomedicals, Santa Ana, USA) following manufacture's recommendations. 16S rRNA gene was amplified by PCR using primers 27F (5′-AGAGTTTGATCCTGGCTCAG-3′) and 1492R (5′-GGTTACCTTGTTACGACTT-3′) and the amplicons were sequenced using Sanger sequencing by an external service provider (Eurofins Genomics, Germany). Obtained nucleotide sequences were compared by BLAST against the NCBI’s targeted loci project for the 16S ribosomal RNA based on NCBI’s non-redundant nucleotide database [RefSeq]^29^. The BLAST similarity search of 16S rRNA gene sequences indicated that strain SubTr2 was closely related to *Pseudoalteromonas rubra* ATCC 29570 (99.55% identity). *Escherichia coli* DH5α (Thermo Fisher Scientific, Waltham, MA, USA) is a non-antibiotic producing laboratory strain commonly used in molecular studies and antimicrobial test methods.

The antimicrobial activity of SubTr2 was evaluated according to the agar well diffusion assay. One ml of SubTr2 strain culture, grown for 24 h at 27 °C in Bertani broth (LB), was centrifuged at 14,000*g* for 5 min and supernatant was filter-sterilized through a 0.22 µm polycarbonate membrane (Merck Millipore, Burlington, MA, USA). Tryptic soy agar plates (TSA) or yeast extract peptone dextrose agar (YPDA) plates were swabbed on the surface with cultures of *E. coli*, *Staphylococcus aureus*, *Listeria innocua* and *Candida albicans*. Then, 5 mm diameter wells were made in the agar media and 30 µl of supernatants were added in the wells. After incubation at 37 °C for 24 h, all plates were examined for the presence of zone of inhibition around the wells.

### Preparation of heat-killed biomass

For microbial biomass production, SubTr2 was grown in Difco™ Marine Broth 2216 (MB; Thermo-Fisher Scientific, Waltham, MA, USA) at 25 °C and DH5α was grown in LB medium (Thermo Fisher Scientific, Waltham, MA, USA) at 37 °C. Strains were grown under constant shaking at 150 rpm until they reached their stationary growth phase (72 h for SubTr2 and 24 h for *E. coli* DH5α). Bacterial biomass of each strain was harvested by centrifugation at 2500*g* for 1 h at 4 °C. The supernatant was discarded, and the biomass pellet was repeatedly resuspended (3×) in sterile ASW and then harvested by centrifugation. This washing step was preform to minimize residual nutrient carryover. The pellet obtained for each strain was then incubated in a water bath at 90 °C for 5 min, as previously described^30^. An aliquot of the pellet of each strain was streaked onto agar plates containing their respective culture media before and after the heating step in order to evaluate their purity and viability. These plates were inspected for growth every 24 h for 5 days. No growth was detected in plates inoculated with the heat-killed biomass, indicating that the procedure was effective. Inactivated SubTr2 (Subt) and DH5α (Ecol) biomasses were then lyophilized during 24 h and stored at 4 °C until use. After lyophilization, the 16S rRNA gene sequences of both strains were obtained and their identity confirmed as previously described.

### Experimental approach and sampling

Due to the uncertainties of the potential effects of the heat-killed biomass on water quality (e.g., increased nutrient levels, microbial load and potential pathogens) and fish health, we used an in vitro model to test our hypotheses without adversely affecting fish welfare. The water used throughout our experiments was collected once from a RAS for the co-culture of european seabass (*Dicentrarchus labrax*) and gilthead seabream (*Sparus aurata*) at RiaSearch Lda (Murtosa, Portugal) and immediately transferred to the laboratory. The RAS water was distributed in 250 ml Erlenmeyer flasks (50 ml/flask) and freeze-dried inactivated *E. coli* DH5α (Ecol) or *P. rubra* SubTr2 (Subt) were added [0.2% (w/v)]. The controls (Cont) for the in vitro experiment consisted of four flasks containing RAS water without addition of heat-killed biomass. Four replicates were used for each treatment. The flasks were placed in an orbital shaker and incubated in the dark at 21 °C (RAS water temperature) under constant agitation (150 rpm) for 3 days. Twenty percent (v/v) of the water from each treatment was replaced with filter-sterilised RAS water containing 0.2% (w/v) of respective inactivated bacterial biomass (Subt and Ecol), or water with no inactivated biomass (Cont), at 24 and 48 h after the beginning of the experiment. The strategy of using partial water replacement and three days of incubation was adopted to minimize water quality deterioration. The RAS water quality is typically maintained by filtration devices (e.g., biofilters and mechanical filters), ozonation and UV treatments and partial water changes (~ 20% v/v per day for the RAS sampled in this study).

At the end of the experiment 1 ml of each flask was collected for flow cytometry analysis and the remained volume was filtered through a 0.22 μm polycarbonate membrane (Merck Millipore, Burlington, MA, USA). The filtrate was collected for physicochemical analysis and the filter membranes were immersed in absolute ethanol and stored at − 20 °C until DNA extraction (bacterioplankton analysis).

### Flow cytometry and physicochemical analyses

Flow cytometry analyses were performed using the commercial kit LIVE/DEAD BacLight Bacterial Viability and Counting Kit (ThermoFisher Scientific, Waltham, MA, USA) according to the manufacturer’s instructions, and using an Attune^®^ Acoustic Focusing Cytometer (Applied Biosystems, Foster City, CA, USA). Bacterial cell concentrations were considered as all events in flow cytometry graphs (no gating was applied) and results were expressed as events*µL^−1^. Ammonia/ammonium, nitrite, and nitrate were quantified by visible light spectrometry, using colorimetric methods appropriate for seawater samples (Spectroquant-Merck, Kenilworth, NJ, USA). Concentrations were calculated from measured absorbance based on linear regression of calibration curves [NO_3_^−^ (R^2^ = 0.9979); NO_2_^−^ (R^2^ = 0.9953); NH_3_/NH_4_^+^ (R^2^ = 0.9958)]. Dissolved organic carbon (DOC) was quantified to evaluate the effect of modulator addition on the organic carbon content of the system. This analysis was performed using infrared detection [SM 5310] at A3lab (Ilhavo, Portugal). pH was measured using a calibrated Consort C932 electrochemical analyser (Consort BVBA, Turnhout, Belgium).

### DNA extraction and high-throughput sequencing

Filter membranes were cut into small pieces and DNA was extracted using the FastDNA SPIN Kit for Soil (MP Biomedicals, Santa Ana, USA) following the manufacturer’s instructions. For high-throughput sequencing, the hypervariable region V4 of the 16S rRNA gene was PCR-amplified using the primers 515F (GTGCCAGCMGCCGCGGTAA) and 806R (GGACTAHVGGGTWTCTAAT) as previously described^31^. Library preparation and sequencing were performed in a MiSeq sequencing platform at the Molecular Research LP (www.mrdnalab.com; Shallowater, TX, USA), following standard Illumina procedures (Illumina, San Diego, CA, USA).

### Bacterioplankton composition and statistical analyses

QIIME2 (version 2020.8) was used to analyse the 16S rRNA gene amplicon libraries^32^. The DADA2 plugin^33^ was used to trim sequences (final length 205 bp), which were demultiplexed and used to generate the amplicon sequence variants (ASV) abundance table. Taxonomy was assigned to ASVs using a sci-kit-learn Naïve Bayes classifier^34^, based on the SILVA database for the amplified region (V4) (version 138.1, released August 27, 2020) at 99% similarity. The classifier is available at https://docs.qiime2.org/2020.8/plugins/; accessed 17/11/2020.

The ASV abundance table obtained with QIIME2 was imported into R (R Project for Statistical Computing, Vienna, Austria) for removal of singletons, chloroplasts, mitochondria, and other non-bacterial ASVs. All ASVs with > 99% similarity to 16S sequences of *P. rubra* SubTr2 (MK533559) and *E. coli* DH5α (CP026085.1:465734–467287) were also removed from the library. Alignments were performed using the BLAST algorithm.

Richness, evenness (Pielou's J), Fisher's alpha and Shannon's H' diversity indices were obtained using the rarefy() and diversity() functions in vegan^35^. We tested for significance differences in community diversity with an analysis of deviance using the glm() function in the stats package in R^36^. A number of these variables included an excess of zero counts in the samples, therefore, we set the family argument to ‘tweedie’ using the tweedie function in R with var.power = 1.5 and link.power = 0 (a compound Poisson – gamma distribution).

For compositional analyses, the ASV table in the present report was transformed using the ordinate() function in the phyloseq package^37^. First, a phyloseq object was generated using the phyloseq() function; input for the function included the ASV table, taxonomic metadata for the ASV table and metadata for each sample. For the ordination analysis we rarefied the ASV table with the rarefy_even_depth() function. The ordinate() function in the phyloseq package was subsequently used with the phyloseq object as input, the method argument set to 'PCoA' and the distance argument set to 'bray'. A biplot was then produced using the plot_ordination() function with the type argument set to 'biplot'. To test for significant differences between treatments, permutational analysis of variance (PERMANOVA) was performed, using the adonis() function of the vegan package^35^. The Bray–Curtis distance matrix was used as the response variable, and the treatment as independent variable. The number of permutations was set to 999.

Environmental parameters were fit onto PCO ordinations of ASV composition using the envfit() function in vegan. Using the envfit() function, we also tested for significant relationships between these variables and ASV ordination using 999 permutations; all other arguments in the function were left as default. Dissolved organic carbon (DOC), flow cytometry (microbial abundance, Cyto) and NH_3_/NH_4_^+^ were found to be significantly associated with the ASV ordination. We then tested for significant differences between treatments in these parameters. We checked for deviations from normality in DOC, flow cytometry results and NH_3_/NH_4_^+^ concentrations with the shapiro() function and tested for homogenous variance with the bartlett.test() function in R. Flow cytometry and NH_3_/NH_4_^+^ concentrations did not deviate significantly from normality and variance also did not differ significantly between treatments. We then tested for significant differences between treatments using analysis of variance (ANOVA) with the aov() function in R. The emmeans() function in the emmeans library was used to perform multiple comparisons of concentration among treatments using estimated marginal means (EMM) with the false discovery rate (fdr) method in the adjust argument of the emmeans() function and a P value of 0.05. DOC concentrations deviated significantly from normality. We, thus, performed a PERMONOVA using the adonis() function in R to test for significant differences in DOC concentrations^35^. In the PERMANOVA, the Euclidean distance matrix (log transformed) of DOC concentrations was the response variable with treatment as independent variable. The number of permutations was set to 999.

The relative abundances of selected prokaryotic higher taxa were tested for significant differences among treatments with an analysis of deviance using the glm() function with the family argument set to quasibinomial. Using the GLM model, we tested for significant variation among factors with the anova() function in R using with the F test. We also used the emmeans function to perform multiple comparisons of mean abundance among treatments as described above.

### Predicted metagenomic analysis

The Tax4Fun2 library^38^ in R was used to predict the metagenome of each sample with the KEGG (Kyoto Encyclopedia of Genes and Genomes) database. First, the runRefBlast function with the database mode was set to “Ref100NR” and the path_to_otus argument set to the representative sequences file generated using QIIME2. The makeFunctionalPrediction argument was then used with the path_to_otu_table argument set to the ASV table and the min_identity_to_reference argument set to 0.95. Default settings were used for the other arguments. The relative abundance of selected pathways was tested for significant differences among treatments with an analysis of deviance using the glm() function with the family argument set to quasibinomial. Using the GLM model, we tested for significant variation among factors with the anova() function in R using with the F test. The emmeans function was used to perform multiple comparisons of mean abundance among treatments.

## Results and discussion

### Evaluation of the antagonistic activity of *Pseudoalteromonas rubra* SubTr2

In this study, the bacterial strain SubTr2 showed antagonistic activity against *S. aureus* in an agar well diffusion assay, but no activity was observed against *E. coli*, *L. innocua* and *C. albicans* (data not shown). SubTr2 is a close phylogenetic relative of *P. rubra* ATCC 29570 (99.55% identity). Like other *Pseudoalteromonas* species, *P. rubra* is known to produce pigments with biotechnological potential^21,22^. The primary pigments produced by *P. rubra* strains include cycloprodigiosin, prodigiosin, 2-methyl-3-hexyl-prodiginine, 2-methyl-3-butyl-prodiginine, and 2-methyl-3-heptyl-prodiginine^39^. These pigments can have several biological functions, including larvicidal, immunomodulatory and antitumoral, anti-nematoid and antimicrobial activities^23^.

### Bacterioplankton community structure and physicochemical parameters

**Figure 1:**
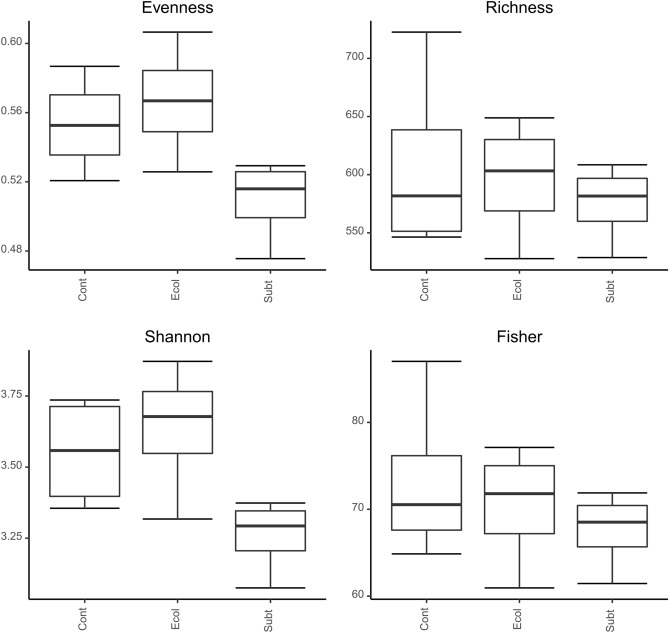
Boxplots of selected diversity indices. Results of GLM-ANOVA analyses: Evenness: F_2,9_ = 4.3, P = 0.049; Richness: F_2,9_ = 0.32, P = 0.734; Shannon: F_2,9_ = 4.38, P = 0.047; Fisher: F_2,9_ = 0.59, P = 0.573. *Cont:* Control; *Ecol:* *E. coli* DH5α; *Subt:* *P. rubra* SubTr2.

**Figure 2:**
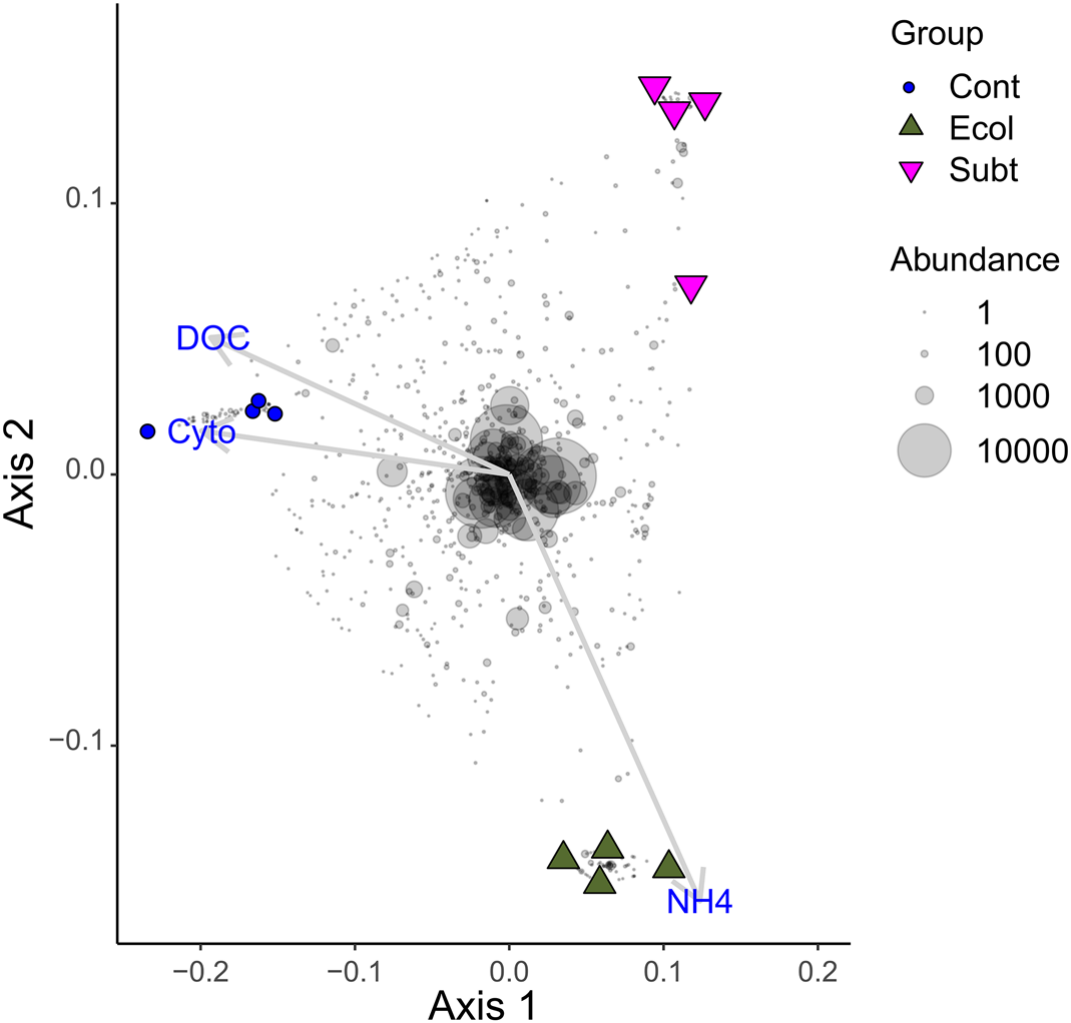
Ordination showing the first two axes of the principal coordinates analysis (PCO) of ASV composition. Symbols are colour coded and represent samples from different treatments as shown in the legend on the right side of the figure. Grey symbols represent weighted averages scores for ASVs. The symbol size is proportional to group abundance (number of sequence reads). The legend symbols represent the following groups: *Cont:* Control; *Ecol:* *E. coli* DH5α; *Subt:* *P. rubra* SubTr2. Only significant vectors are represented.

The high throughput 16S rRNA gene amplicon sequencing of bacterioplankton communities investigated in this study resulted in 5239205 sequences assigned to 2370 ASVs. Evenness and Shannon’s H’ diversity indices were significantly lower in the *P. rubra* SubTr2 treatment (Subt) than in both other treatments (Ecol and Cont; Fig. 1; Table S1). In contrast, there were no significant differences in Richness and Fisher’s α diversity indices among treatments. Bacterial composition differed significantly (PERMANOVA: F_2,9_ = 10.717, p = 0.001) among treatments as shown in the PCO analysis (Fig. 2). In line with our findings, previous studies have also shown that the supplementation of fish diets with postbiotic biomasses induces compositional shifts in the fish faecal microbiome^17,18^. However, such an effect has not been previously described for bacterioplankton in aquaculture systems.

**Figure 3:**
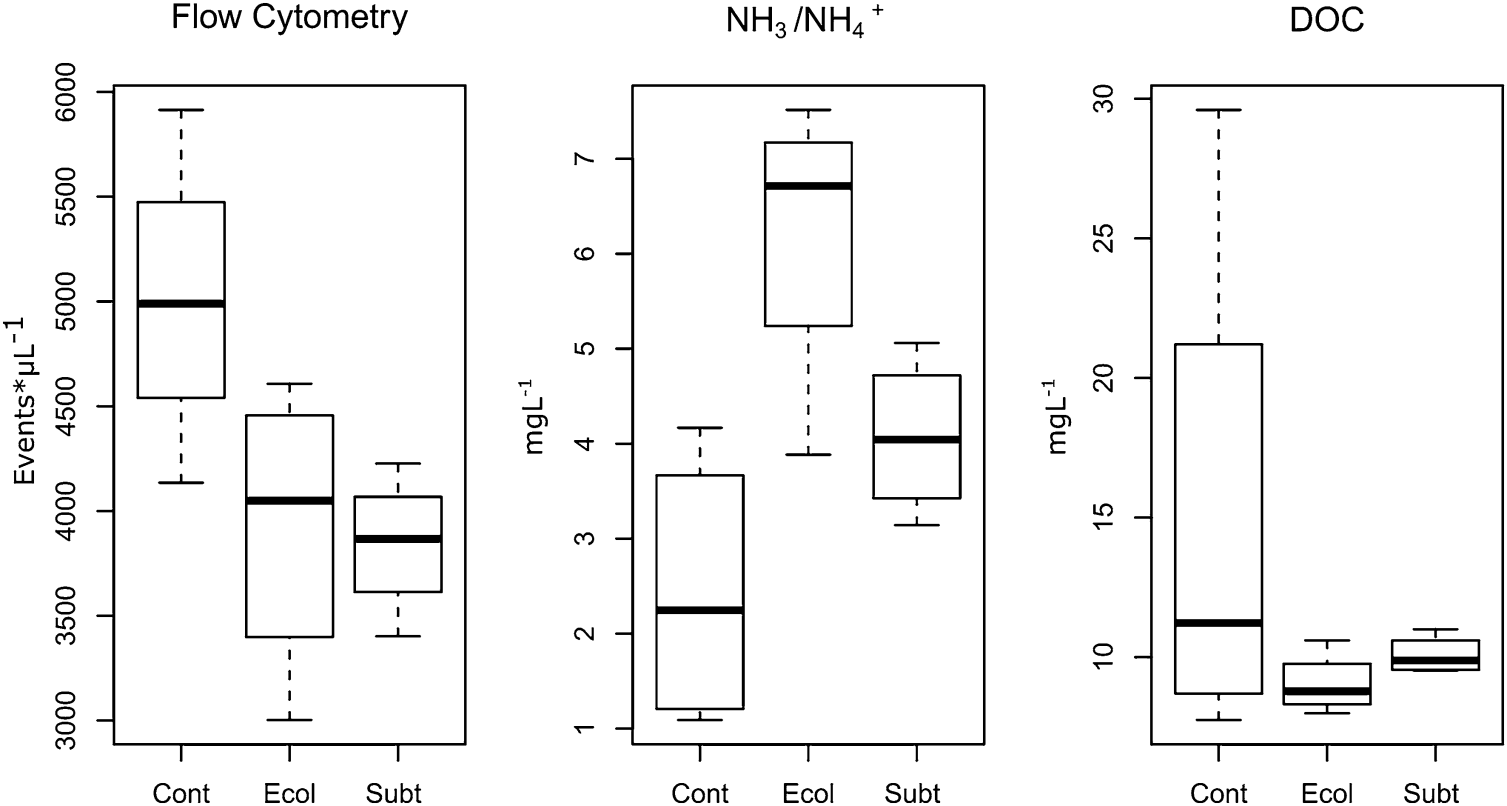
Boxplots of water parameters that were significantly associated with the ASV ordination. *Cont:* Control; *Ecol:* *E*. *coli* DH5α; *Subt:* *P*. *rubra* SubTr2. Flow cytometry-ANOVA: F_2,9_ = 4.46, P = 0.04; NH_3_/NH_4_^+^-ANOVA: F_2,9_ = 7.88, P = 0.01; DOC (Dissolved organic carbon)-PERMANOVA: F_2,9_ = 1.17, P = 0.369.

The DOC, bacterial cell concentrations and ammonia (NH_3_/NH_4_^+^) concentrations were significantly associated with the ASV ordination (Fig. 2; Tables S2, S3). DOC and microbial abundance concentrations were associated with samples from the control treatment (Cont), while ammonia was associated with samples from the *E.coli* DH5α treatment (Ecol). There were no significant associations between NO_2_^−^, NO_3_^−^ or pH and the ASV ordination (Fig. 2; Table S3). In line with these results, bacterial cell and ammonia concentrations varied significantly. Cell concentration was higher in Cont than both Ecol and Subt, while ammonia was significantly higher in Ecol than in Cont (Fig. 3; Table S4). There was no significant difference in ammonia concentration between Subt and Cont (Table S4). According to the ecological theory of r/K selection, a nutrient-rich environment should select for fast growing, opportunistic, r-strategist microbial populations and increase microbial abundance. However, in contrast to this expectation, the microbial abundance and DOC concentrations were lower in the Subt and Ecol treatments (although this was non-significant for DOC) despite the bacterial biomass from both species being potential nutrient sources. Such an effect is intriguing and may have important implications for controlling water microbial loads in RAS. However, it is important to highlight that potential antagonistic substances released by *P. rubra* SubTr2 biomass may have also played a role in reducing microbial abundance in our experiment.

### Higher taxon abundance

**Figure 4:**
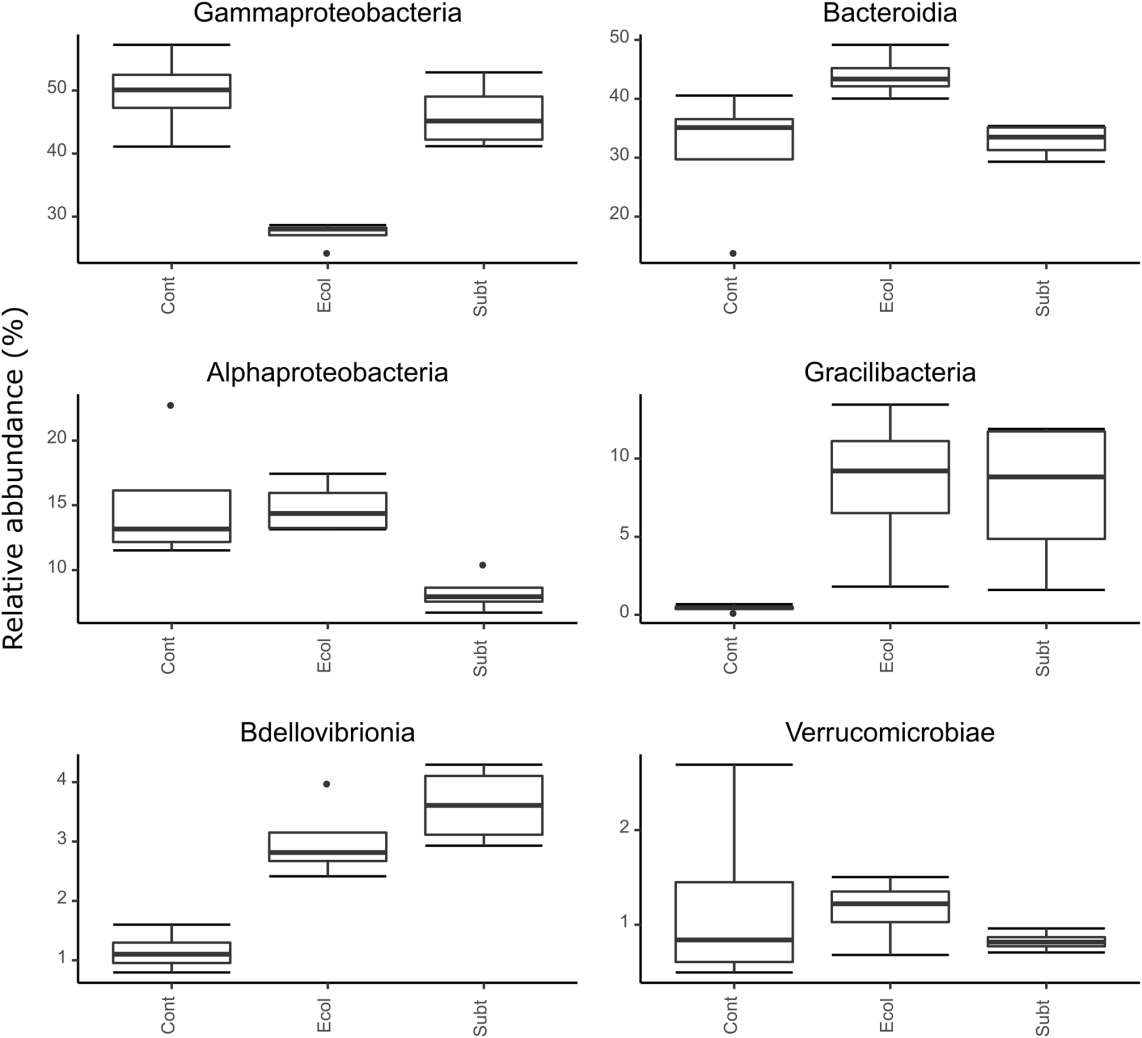
Relative abundances of the six most abundant prokaryotic classes. Results of GLM-ANOVA analyses Gammaproteobacteria: F_2,9_ = 5.91, P = 0.023; Bacteroidia: F_2,9_ = 8.84, P = 0.008; Alphaproteobacteria: F_2,9_ = 1.1, P = 0.375; Gracilibacteria: F_2,9_ = 17.21, P < 0.001; Bdellovibrionia: F_2,9_ = 52.29, P < 0.001; Verrucomicrobiae: F_2,9_ = 0.05, P = 0.949. The groups sampled were: *Cont:* Control; *Ecol:* *E. coli DH5α*; *Subt:* *P. rubra* SubTr2.

The relative abundance of Gammaproteobacteria was significantly lower in the *E.coli* DH5α treatment than the *P. rubra* SubTr2 treatment (Fig. 4; Table S5). On the contrary, the relative abundance of Bacteroidia was highest in the *E.coli* DH5α treatment (Fig. 4; Table S5). Members of both classes are ubiquitous in marine environments and are often described as key players in the degradation of marine organic matter^40,41^. The relative abundances of Gracilibacteria and Bdellovibrionia were significantly higher in the *E.coli* DH5α and *P. rubra* SubTr2 treatments than the control treatment (Fig. 4; Table S5). Gracilibacteria belongs to the candidate phyla radiation (CPR), which are known to have limited biosynthetic capabilities but have been shown to have a versatile metabolic ability for nutrient cycling (e.g., carbon, hydrogen, and, possibly, sulphur and nitrogen)^42,43^. The enrichment of Bdellovibrionia may have played a role in reducing the bacterial load of the RAS water. This class consists of obligate predators (*Bdellovibrio* and like organisms—BALOs), which prey upon a broad range of Gram-negative bacteria^44^. Previous studies also showed that BALOs are effective predators of planktonic and biofilm-associated cells, which may also play a role in reducing water bacterial load^45–47^.

**Figure 5:**
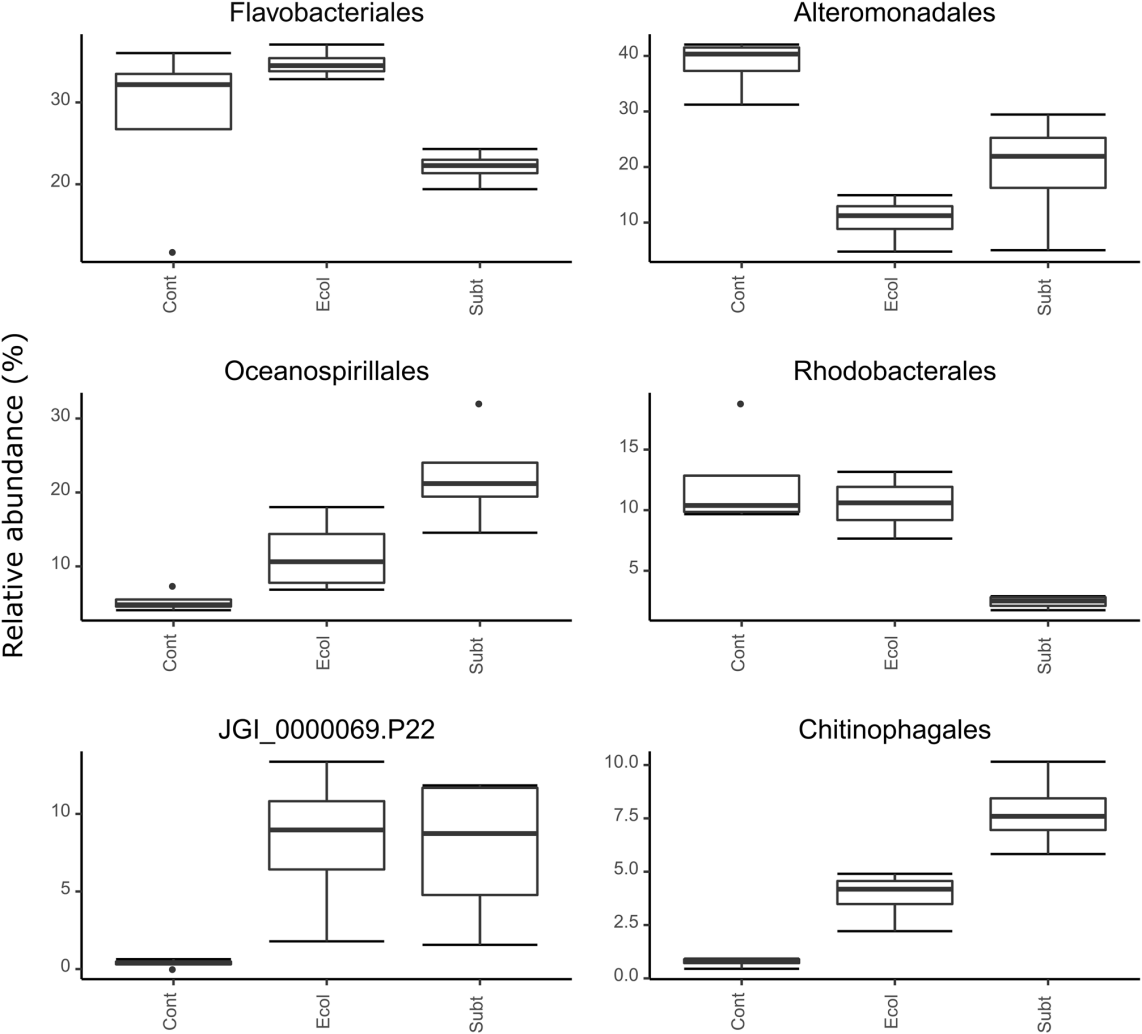
Relative abundances of the six most abundant prokaryotic orders. Results of GLM-ANOVA anaylsis: Flavobacteriales: F_2,9_ = 4.69, P = 0.04, Alteromonadales: F_2,9_ = 6.12, P = 0.021, Oceanospirillales: F_2,9_ = 11.76, P = 0.003, Rhodobacterales: F_2,9_ = 8.64, P = 0.008, JGI_0000069.P22: F_2,9_ = 16.77, P < 0.001, Chitinophagales: F_2,9_ = 65.73, P < 0.001. The groups sampled were: *Cont:* Control; *Ecol*: *E. coli* DH5α; *Subt:* *P. rubra* SubTr2.

At the order level, the relative abundances of Oceanospirillales, Chitinophagales and candidate division JGI_0000069.P22 (Gracilibacteria) were significantly higher in the *E.coli* DH5α and *P. rubra* SubTr2 treatments than the control treatment (Fig. 5; Table S6). In contrast, the relative abundance of Alteromonadales was significantly lower in both treatments compared to the control while the relative abundance of Rhodobacterales was significantly lower in the *P. rubra* SubTr2 treatment when compared to both other treatments (Fig. 5; Table S6). Oceanospirillales are ubiquitous in marine environments, including marine aquacultures^48,49^ and consists of bacterial species known to excrete hydrolytic enzymes and emulsifying agents involved in the degradation of aquatic organic matter^50^. Chitinophagales are an important constituent of marine bacterioplankton, particularly in pelagic zones^51^. Members of this order are known for their ability to catabolise complex polysaccharides (e.g., starch, cellulose, xylans, pectins and chitin) and proteins^52,53^. Furthermore, some Chitinophagales species have been shown to produce several secondary metabolites, including antimicrobial and antifungal compounds, which could have interesting antagonistic activities against bacterial pathogens^54,55^. Alteromonadales and Rhodobacterales are also known as key players in the process of degradation of algal-derived polysaccharides in marine environments^56^.

### In-depth analysis of bacterioplankton ASVs

**Figure 6:**
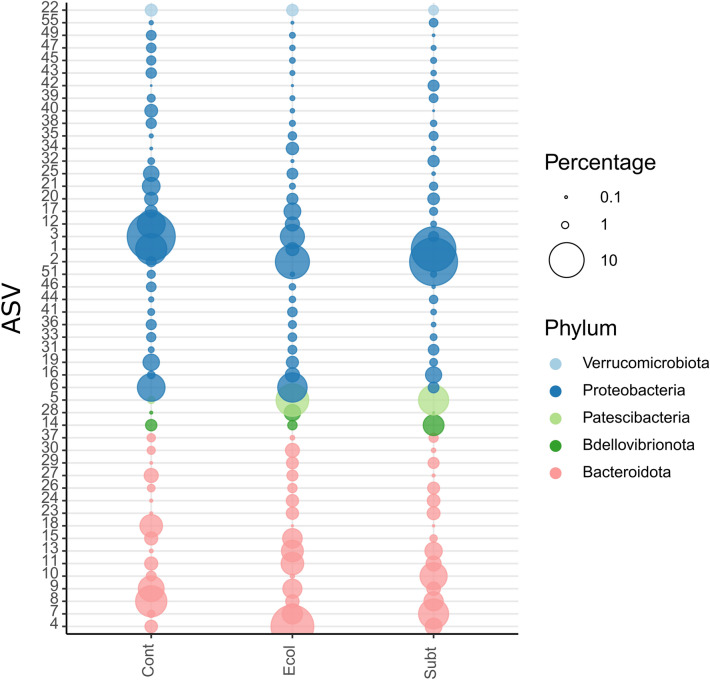
Relative abundances of the 50 most abundant ASVs colour-coded according to prokaryotic phylum for: *Cont:* Control; *Ecol:* *E. coli* DH5α; *Subt:* *P. rubra* SubTr2. The circle size of the ASV is proportional to the mean percentage of sequences per treatment as indicated by the symbol legend.

The 50 most abundant ASVs are shown in Fig. 6 and Table S7 highlighting marked differences in the abundances of certain ASVs among treatments. A number of ASVs, for example, were more abundant in *E.coli* DH5α and *P. rubra* SubTr2 treatments when compared to the control. This included ASVs 2, 5 and 13 assigned to the genus *Oceanospirillum* (*O. beijerinckii*), candidate division JGI_0000069.P22 (Gracilibacteria) and Flavobacteriaceae family, respectively (Fig. 6). The *Oceanospirillum* genus lacks the ability to catabolize carbohydrates, but may be involved in the degradation of hydrocarbons in marine environments^57,58^. Gracilibacteria members have also been implicated in nutrient cycling^42,43^. Planktonic Flavobacteria can actively exploit polymeric organic substrates (e.g., carbohydrates, polypeptides, and lipids) derived from marine detritus^59^.

Certain ASVs were also enriched in single treatments. ASVs 3 and 12, for example, both assigned to the genus *Glaciecola*, were enriched in the control treatment (Table S7)^60^. Previous studies suggested that members of this genus may play a key role in the breakdown of complex dissolved organic matter derived from terrestrial environments and phytoplankton blooms in seawater^61,62^. ASV 4, assigned to the genus *Tenacibaculum*, in turn, was enriched in the *E. coli* DH5α treatment. *Tenacibaculum* members have been identified as the aetiological agents responsible for marine tenacibaculosis (ulcerative disease) in fish aquacultures^63^. ASV 10, assigned to the NS9_marine_group (Flavobacteriales order), was enriched in the *P. rubra* SubTr2 treatment.

In contrast to the above, ASVs 6, 15 and 27, assigned to the genera *Yoonia-Loktanella*, *Tenacibaculum* and *Luteibaculum*, respectively, were depleted in the *P. rubra* SubTr2 treatment compared to both other treatments. Members of the *Yoonia*-*Loktanella* group, a constituent of the paraphyletic Roseobacter clade^64^, are equipped with a large metabolic repertory, which allows them to colonize a range of marine habitats and form symbiotic interactions^65^. A previous study published by Bakenhus et al.^41^ highlighted the importance of members of the Roseobacter clade and Bacteroidota in processing phytoplankton-derived organic matter in seawater. The genus *Luteibaculum,* includes *L. oceani*, a carotenoid producing bacteria^66^.

ASVs 14 and 28, both assigned to the Bacteriovoracaceae family and genus *Peredibacter*, were differentially enriched in the *P. rubra* SubTr2 and *E.coli* DH5α treatments, respectively. The genus *Peredibacter* consists of bacterial predators of bacteria belonging to the BALO group (Fig. 6; Table S7)^67,68^. The BALO are a group of obligate predatory prokaryotes thought to prey exclusively on gram-negative bacteria^44^. BALO isolated from fishponds have been reported to contribute to fish health and growth performance, by reducing the incidence of diseases caused by *Aeromonas hydrophila* and *V. alginolyticus*^69^. Recent studies have attempted to control bacterial pathogens in aquaculture systems by using BALO cultures to increase their abundance in the rearing water^70,71^. However, some bioaugmentation strategies are thought to be ineffective due to the maladaptation of allochthonous inoculums to the new environment, where local abiotic conditions and the resident microbial community may impede their establishment and proliferation^72,73^.

### Predicted metagenomic analysis

**Figure 7:**
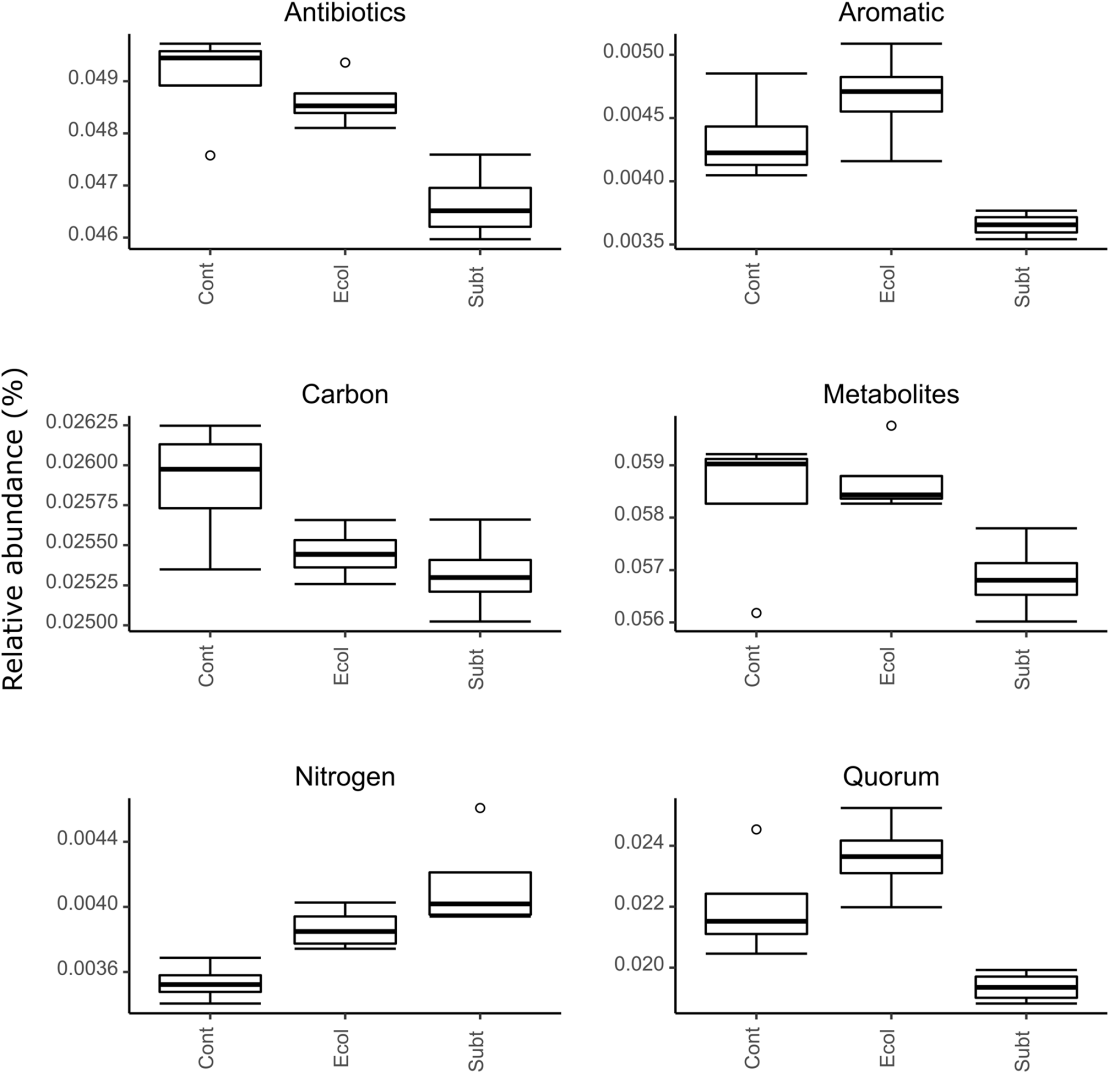
Variation in the relative gene count abundances of selected KEGG level 1 categories: antibiotics: biosynthesis of antibiotics; aromatic: degradation of aromatic compounds; carbon: carbon metabolism; metabolites: biosynthesis of secondary metabolites; nitrogen: nitrogen metabolism; quorum: quorum sensing. The groups sampled were: *Cont:* Control, *Ecol:*  *E. coli* DH5α, *Subt:* *P. rubra* SubTR1. Results of the GLM-ANOVA analyses: antibiotics: F_2,9_ = 11.37, P = 0.003; aromatic: F_2,9_ = 12.53, P = 0.003; carbon: F_2,9_ = 4.24, P = 0.05; metabolites: F_2,9_ = 3.78, P = 0.064; nitrogen: F_2,9_ = 9.42; P = 0.006, quorum: F_2,9_ = 11.48, P = 0.003.

In the present study, the predictive metagenomic analysis focused on KEGG level 1 categories related to relevant functions to aquaculture systems (e.g., bacterial colonization, nutrient cycling and biosynthesis of secondary metabolites). The predicted relative gene counts of the carbon metabolism were lower and the nitrogen metabolism higher in the *P. rubra* SubTr2 and *E.coli* DH5α treatments than the control (Fig. 7; Table S8). This result was in line with the DOC and ammonia/ammonium results in both treatments (Fig. 3). The predicted relative gene counts of the quorum sensing category were also significantly lower in the *P. rubra* SubTr2 treatment than both other treatments (Fig. 7; Table S8). The expression of virulence factors and biofilm formation is often regulated by quorum sensing. Its disruption has been seen as a potential strategy to prevent pathogen infection in aquacultures^74^. However, the gene count abundances of the biosynthesis of antibiotics and biosynthesis of secondary metabolites categories were also lower in the *P. rubra* SubTr2 treatment. Production of antibiotics and other secondary metabolites can provide an advantage to producing strains when competing against other microorganisms for the same ecological niches^75^.

## Conclusion

The results obtained in this study suggest that heat-killed microbial biomasses can have generalised and species-specific effects on the structure and function of aquaculture bacterioplankton communities. Our results, furthermore, showed that under laboratory-controlled conditions these biomasses have the potential to increase the abundance of bacterial predators, thereby potentially reducing bacterial load in addition to influencing nutrient cycling and bacterial pathogen abundance in aquaculture water. The results based on metagenomic prediction suggest potentially beneficial (reduction in quorum sensing) and potentially adverse (reduction in antibiotic production) effects of *P. rubra* SubTr2 supplementation to aquaculture water. Together, despite its preliminary nature, for the first time, this study showed that heat-killed microbial biomass has potential application as an in situ modulator of aquaculture bacterioplankton communities. Furthermore, our findings raise interesting questions about the use of heat-killed *P. rubra* SubTr2 and other microbial strains as microbiome modulators in RAS water: (1) What are the effects of non-viable microbial biomasses (e.g., probiotic and non-probiotic strains) on bacterioplankton structure and function and water quality during fish production? (2) Are their microbiome modulatory effects extended to other aquaculture compartments (e.g., biofilm, fish gill, gut and mucus)? (3) What are their effects on fish health and are these effects comparable to the health benefits conferred by postbiotics in fish feed? (4) Can non-viable microbial biomasses be used to stimulate the development of BALO (“living antibiotics”) to control the development of fish pathogens and reduce water bacterial load in aquaculture systems? Further research is needed under experimental and commercial aquaculture settings to answer the questions raised in this study.

## Supplementary Information

Supplementary Information.

## Data Availability

Sequences used in this study were uploaded to the NCBI ShortRead Archive (BioProject PRJNA851239; Biosamples SRR19790184–SRR19790196). The 16S sequence of strain SubTr2 is deposited at NCBI Genbank (accession no. MK533559).
