# Supplementary material for: In vitro study of the modulatory effects of heat-killed bacterial biomass on aquaculture bacterioplankton communities
Source: Sci Rep. 2022 Nov 16;12:19699. doi: 10.1038/s41598-022-23439-8 (PMC9669034; doi:10.1038/s41598-022-23439-8)
Supplement: Supplementary file 1 — Supplementary Information. [file 41598_2022_23439_MOESM1_ESM.docx]

*In vitro* study of the modulatory effects of heat-killed bacterial biomass on aquaculture bacterioplankton communities

Sousa, J.M.G.^a∆^ Louvado, A.^a∆^; Coelho, FJRC^a^; Oliveira, V. ^a^, Oliveira, H.^a^, Cleary, D.F.R.^a^; Gomes, N.C.M.^a*^

^a^CESAM - Centre for Environmental and Marine Studies, Department of Biology, University of Aveiro, 3810-193 Aveiro, Portugal

^∆^both authors share first authorship

*corresponding author

Table S1: Emmeans results for selected diversity indices.

| id | Treatments | estimate | SE | z.ratio | p.value |
| --- | --- | --- | --- | --- | --- |
| Evenness | Cont - Ecol | -0.02379 | 0.037626 | -0.63218 | 0.52727 |
| Evenness | Cont - SubT | 0.082847 | 0.038134 | 2.172515 | **0.044725** |
| Evenness | Ecol - SubT | 0.106633 | 0.038024 | 2.804391 | **0.015124** |
| Richness | Cont - Ecol | 0.020481 | 0.070099 | 0.292173 | 0.770154 |
| Richness | Cont - SubT | 0.055796 | 0.070412 | 0.792414 | 0.770154 |
| Richness | Ecol - SubT | 0.035315 | 0.070591 | 0.500272 | 0.770154 |
| Shannon | Cont - Ecol | -0.02344 | 0.038403 | -0.61041 | 0.541588 |
| Shannon | Cont - SubT | 0.086155 | 0.038937 | 2.212699 | **0.040378** |
| Shannon | Ecol - SubT | 0.109597 | 0.038826 | 2.822801 | **0.014282** |
| Fisher | Cont - Ecol | 0.039323 | 0.073274 | 0.536649 | 0.59151 |
| Fisher | Cont - SubT | 0.080224 | 0.073656 | 1.089174 | 0.59151 |
| Fisher | Ecol - SubT | 0.040901 | 0.074013 | 0.552621 | 0.59151 |

Table S2: Mean values and standard deviation of nitrites (NO_2_^-^). nitrates (NO_3_^-^). ammonia/ammonium (NH3/NH4^+^). pH. dissolved organic carbon (DOC) and flow cytometry (Cyto).

| **Sample** | **NO_2_^-^ (mg/l)** | **NO_3_^-^ (mg/l)** | **NH_3_/NH_4_^+^** **(mg/l)** | **pH** | **DOC (mg/l)** | **Cyto (events/µL^-1^)** |
| --- | --- | --- | --- | --- | --- | --- |
| **Control** | 0.29 ± 0.07 | 176.85 ± 2.00 | 2.44 ± 1.57 | 7.74 ± 0.45 | 14.95 ± 11.44 | 5006.934 ± 890.97 |
| **SubT** | 0.42 ± 0.05 | 176.52 ± 2.47 | 4.07 ± 0.83 | 7.92 ± 0.08 | 10.07 ± 0.69 | 3840.40 ± 339.69 |
| **Ecol** | 0.40 ± 0.02 | 173.47 ± 10.02 | 6.20 ± 1.60 | 7.74 ± 0.52 | 9.03 ± 1.11 | 3927.02 ± 701.76 |

Table S3: Results of envfit analysis for ordination of first and second axes.

|  | **Axis1** | **Axis2** | **R^2^** | **P** |
| --- | --- | --- | --- | --- |
| NO_3_ | -0.605 | 0.796 | 0.108 | 0.722 |
| NH_3_/NH_4_^+^ | 0.631 | -0.776 | 0.545 | **0.034** |
| pH | 0.792 | 0.61 | 0.114 | 0.601 |
| DOC | -0.971 | 0.237 | 0.336 | **0.037** |
| Cyto | -0.999 | 0.054 | 0.523 | **0.036** |
| NO2 | 0.999 | -0.033 | 0.394 | 0.095 |

Table S4: Emmeans results for selected diversity indices for flow cytometry (Cyto) and dissolved organic carbon (DOC).

| id | Treatments | estimate | SE | t.ratio | p.value |
| --- | --- | --- | --- | --- | --- |
| Cyto | Cont - Ecol | 1079.9 | 435 | 2.480 | **0.0525** |
| Cyto | Cont - SubT | 1166.5 | 435 | 2.679 | **0.0525** |
| Cyto | Ecol - SubT | 86.6 | 435 | 0.199 | 0.8467 |
| NH3/NH4^+^ | Cont - Ecol | -3.77 | 0.952 | -3.960 | **0.0099** |
| NH3/NH4^+^ | Cont - SubT | -1.64 | 0.952 | -1.718 | 0.1199 |
| NH3/NH4^+^ | Ecol - SubT | 2.13 | 0.952 | 2.242 | 0.0755 |

Table S5: Emmeans results for selected bacterial classes.

| id | Treatments | estimate | SE | z.ratio | p.value |
| --- | --- | --- | --- | --- | --- |
| Gammaproteobacteria | Cont - Ecol | 0.410487 | 0.204241 | 2.00982 | 0.066675 |
| Gammaproteobacteria | Cont - SubT | -0.27052 | 0.187296 | -1.44434 | 0.148643 |
| Gammaproteobacteria | Ecol - SubT | -0.68101 | 0.198357 | -3.43323 | **0.001789** |
| Bacteroidia | Cont - Ecol | -0.62275 | 0.153694 | -4.05186 | **0.000152** |
| Bacteroidia | Cont - SubT | -0.5011 | 0.155718 | -3.21798 | **0.001936** |
| Bacteroidia | Ecol - SubT | 0.121649 | 0.143527 | 0.847568 | 0.396678 |
| Alphaproteobacteria | Cont - Ecol | -0.15762 | 0.273612 | -0.57606 | 0.564575 |
| Alphaproteobacteria | Cont - SubT | 0.260417 | 0.288483 | 0.902709 | 0.55002 |
| Alphaproteobacteria | Ecol - SubT | 0.418034 | 0.283327 | 1.475445 | 0.420279 |
| Gracilibacteria | Cont - Ecol | -3.34582 | 0.642958 | -5.20379 | **3.55E-07** |
| Gracilibacteria | Cont - SubT | -3.32541 | 0.643479 | -5.16786 | **3.55E-07** |
| Gracilibacteria | Ecol - SubT | 0.020412 | 0.362399 | 0.056325 | 0.955083 |
| Bdellovibrionia | Cont - Ecol | -1.22013 | 0.163112 | -7.48034 | **1.11E-13** |
| Bdellovibrionia | Cont - SubT | -1.59271 | 0.158156 | -10.0705 | **2.24E-23** |
| Bdellovibrionia | Ecol - SubT | -0.37258 | 0.130922 | -2.84579 | **0.00443** |
| Verrucomicrobiae | Cont - Ecol | -0.05519 | 0.459559 | -0.1201 | 0.904407 |
| Verrucomicrobiae | Cont - SubT | 0.094181 | 0.468263 | 0.201129 | 0.904407 |
| Verrucomicrobiae | Ecol - SubT | 0.149372 | 0.465142 | 0.321133 | 0.904407 |

Table S6: Emmeans results for selected bacterial orders

| id | Treatments | estimate | SE | z.ratio | p.value |
| --- | --- | --- | --- | --- | --- |
| Flavobacteriales | Cont - Ecol | -0.49859 | 0.165172 | -3.01858 | **0.007619** |
| Flavobacteriales | Cont - SubT | -0.2068 | 0.170759 | -1.21103 | 0.225883 |
| Flavobacteriales | Ecol - SubT | 0.29179 | 0.160548 | 1.817465 | 0.103719 |
| Alteromonadales | Cont - Ecol | 1.14387 | 0.329536 | 3.471147 | **0.001555** |
| Alteromonadales | Cont - SubT | 0.337855 | 0.292522 | 1.154976 | 0.2481 |
| Alteromonadales | Ecol - SubT | -0.80601 | 0.340299 | -2.36854 | **0.026787** |
| Oceanospirillales | Cont - Ecol | -0.99864 | 0.403758 | -2.47336 | **0.014406** |
| Oceanospirillales | Cont - SubT | -1.7845 | 0.378172 | -4.71874 | **7.12E-06** |
| Oceanospirillales | Ecol - SubT | -0.78586 | 0.321154 | -2.44699 | **0.014406** |
| Rhodobacterales | Cont - Ecol | -0.02359 | 0.288136 | -0.08188 | 0.934742 |
| Rhodobacterales | Cont - SubT | 1.293557 | 0.348546 | 3.711296 | **0.000309** |
| Rhodobacterales | Ecol - SubT | 1.31715 | 0.347843 | 3.786624 | **0.000309** |
| JGI_0000069.P22 | Cont - Ecol | -3.51725 | 0.690778 | -5.09173 | **5.93E-07** |
| JGI_0000069.P22 | Cont - SubT | -3.50472 | 0.691097 | -5.07125 | **5.93E-07** |
| JGI_0000069.P22 | Ecol - SubT | 0.012529 | 0.375092 | 0.033402 | 0.973354 |
| Chitinophagales | Cont - Ecol | -1.8482 | 0.265391 | -6.96408 | **4.96E-12** |
| Chitinophagales | Cont - SubT | -2.74786 | 0.251362 | -10.9319 | **2.43E-27** |
| Chitinophagales | Ecol - SubT | -0.89966 | 0.181034 | -4.96953 | **6.71E-07** |

Table S7: List of the 50 most abundant ASVs and their taxonomic assignment using the Silva (https://www.arb-silva.de/) database.

| **Abundance** | **ASV** | **Class** | **Order** | **Family** | **Genus** | **Species** |
| --- | --- | --- | --- | --- | --- | --- |
| 476054 | 1 | Gammaproteobacteria | Alteromonadales | Pseudoalteromonadaceae | Pseudoalteromonas | Unassigned |
| 575474 | 2 | Gammaproteobacteria | Oceanospirillales | Oceanospirillaceae | Oceanospirillum | Oceanospirillum_beijerinckii |
| 370799 | 3 | Gammaproteobacteria | Alteromonadales | Alteromonadaceae | Glaciecola | Unassigned |
| 329648 | 4 | Bacteroidia | Flavobacteriales | Flavobacteriaceae | Tenacibaculum | Unassigned |
| 310547 | 5 | Gracilibacteria | JGI_0000069-P22 | JGI_0000069-P22 | JGI_0000069-P22 | uncultured_bacterium |
| 228528 | 6 | Alphaproteobacteria | Rhodobacterales | Rhodobacteraceae | Yoonia-Loktanella | Unassigned |
| 213809 | 7 | Bacteroidia | Chitinophagales | Chitinophagaceae | Edaphobaculum | uncultured_bacterium |
| 196005 | 8 | Bacteroidia | Flavobacteriales | Flavobacteriaceae | Unassigned | Unassigned |
| 154236 | 9 | Bacteroidia | Flavobacteriales | Flavobacteriaceae | Unassigned | Unassigned |
| 133264 | 10 | Bacteroidia | Flavobacteriales | NS9_marine_group | NS9_marine_group | uncultured_Flavobacterium |
| 124573 | 11 | Bacteroidia | Cytophagales | Cyclobacteriaceae | Fabibacter | uncultured_bacterium |
| 120279 | 12 | Gammaproteobacteria | Alteromonadales | Alteromonadaceae | Glaciecola | Unassigned |
| 115413 | 13 | Bacteroidia | Flavobacteriales | Flavobacteriaceae | Unassigned | Unassigned |
| 94192 | 14 | Bdellovibrionia | Bacteriovoracales | Bacteriovoracaceae | Peredibacter | Unassigned |
| 78033 | 15 | Bacteroidia | Flavobacteriales | Flavobacteriaceae | Tenacibaculum | Unassigned |
| 72864 | 16 | Alphaproteobacteria | Rhodospirillales | Terasakiellaceae | uncultured | uncultured_bacterium |
| 61472 | 17 | Gammaproteobacteria | Vibrionales | Vibrionaceae | Vibrio | Unassigned |
| 57643 | 18 | Bacteroidia | Flavobacteriales | Flavobacteriaceae | NS3a_marine_group | Unassigned |
| 55734 | 19 | Alphaproteobacteria | Rhodobacterales | Rhodobacteraceae | Celeribacter | uncultured_Alphaproteobacteria |
| 53658 | 20 | Gammaproteobacteria | Oceanospirillales | Saccharospirillaceae | Oceaniserpentilla | Oceaniserpentilla_haliotis |
| 47128 | 21 | Gammaproteobacteria | Alteromonadales | Colwelliaceae | Colwellia | Unassigned |
| 45198 | 22 | Verrucomicrobiae | Verrucomicrobiales | Rubritaleaceae | Persicirhabdus | uncultured_bacterium |
| 44747 | 23 | Bacteroidia | Flavobacteriales | Cryomorphaceae | Vicingus | Vicingus_serpentipes |
| 44115 | 24 | Bacteroidia | Flavobacteriales | Cryomorphaceae | Vicingus | Unassigned |
| 41315 | 25 | Gammaproteobacteria | Unassigned | Unassigned | Unassigned | Unassigned |
| 36924 | 26 | Bacteroidia | Flavobacteriales | Cryomorphaceae | Owenweeksia | uncultured_marine |
| 36805 | 27 | Bacteroidia | Flavobacteriales | Cryomorphaceae | Luteibaculum | uncultured_Bacteroidetes |
| 36325 | 28 | Bdellovibrionia | Bacteriovoracales | Bacteriovoracaceae | Peredibacter | uncultured_bacterium |
| 35936 | 29 | Bacteroidia | Flavobacteriales | Cryomorphaceae | Unassigned | Unassigned |
| 33582 | 30 | Bacteroidia | Flavobacteriales | Flavobacteriaceae | Aquimarina | Unassigned |
| 28291 | 31 | Alphaproteobacteria | Sneathiellales | Sneathiellaceae | Sneathiella | uncultured_bacterium |
| 23029 | 32 | Gammaproteobacteria | Cellvibrionales | Cellvibrionaceae | Marinagarivorans | gamma_proteobacterium |
| 22621 | 33 | Alphaproteobacteria | Rhodobacterales | Rhodobacteraceae | Unassigned | Unassigned |
| 22166 | 34 | Gammaproteobacteria | Alteromonadales | Colwelliaceae | Thalassotalea | uncultured_bacterium |
| 20357 | 35 | Gammaproteobacteria | Oceanospirillales | Marinomonadaceae | Marinomonas | Marinomonas_communis |
| 20168 | 37 | Bacteroidia | Flavobacteriales | Flavobacteriaceae | Flavobacterium | Flavobacterium_jumunjinense |
| 19484 | 36 | Alphaproteobacteria | Rhodobacterales | Rhodobacteraceae | Unassigned | Unassigned |
| 19021 | 38 | Gammaproteobacteria | Nitrosococcales | Methylophagaceae | Methylophaga | Unassigned |
| 18904 | 40 | Gammaproteobacteria | Alteromonadales | Alteromonadaceae | Alteromonas | Unassigned |
| 18695 | 41 | Alphaproteobacteria | Rhizobiales | Devosiaceae | Maritalea | uncultured_marine |
| 18526 | 39 | Gammaproteobacteria | Oceanospirillales | Saccharospirillaceae | Bermanella | uncultured_bacterium |
| 17875 | 42 | Gammaproteobacteria | Pseudomonadales | Moraxellaceae | Paraperlucidibaca | Unassigned |
| 16367 | 43 | Gammaproteobacteria | Oceanospirillales | Nitrincolaceae | Marinobacterium | uncultured_Marinobacterium |
| 16097 | 44 | Alphaproteobacteria | Parvibaculales | Parvibaculaceae | Mf105b01 | uncultured_Alphaproteobacteria |
| 15615 | 45 | Gammaproteobacteria | Oceanospirillales | Pseudohongiellaceae | Pseudohongiella | Unassigned |
| 14460 | 46 | Alphaproteobacteria | Rhodobacterales | Rhodobacteraceae | Pseudophaeobacter | Pseudophaeobacter_arcticus |
| 14383 | 47 | Gammaproteobacteria | Alteromonadales | Alteromonadaceae | Alteromonas | Unassigned |
| 13130 | 49 | Gammaproteobacteria | Pseudomonadales | Pseudomonadaceae | Pseudomonas | Unassigned |
| 12280 | 51 | Alphaproteobacteria | Rhodospirillales | Thalassospiraceae | Thalassospira | Unassigned |
| 11848 | 55 | Gammaproteobacteria | Cellvibrionales | Spongiibacteraceae | Spongiibacter | Unassigned |

Table S8: Emmeans results for selected KEGG pathways.

| id | Treatments | estimate | SE | z.ratio | p.value |
| --- | --- | --- | --- | --- | --- |
| Antibiotics | Cont - Ecol | 0,008835 | 0,011175 | 0,790639 | 0,429154 |
| Antibiotics | Cont - SubT | 0,051616 | 0,011235 | 4,594259 | **1,3E-05** |
| Antibiotics | Ecol - SubT | 0,042781 | 0,011247 | 3,803714 | **0,000214** |
| Aromatic | Cont - Ecol | -0,07286 | 0,048717 | -1,49555 | 0,134771 |
| Aromatic | Cont - SubT | 0,172513 | 0,050254 | 3,432846 | **0,000896** |
| Aromatic | Ecol - SubT | 0,245371 | 0,049821 | 4,925009 | **2,53E-06** |
| Carbon | Cont - Ecol | 0,017274 | 0,007946 | 2,173817 | **0,044578** |
| Carbon | Cont - SubT | 0,023569 | 0,007953 | 2,963667 | **0,00912** |
| Carbon | Ecol - SubT | 0,006295 | 0,00797 | 0,789867 | 0,429606 |
| Metabolites | Cont - Ecol | -0,00591 | 0,01239 | -0,47694 | 0,633404 |
| Metabolites | Cont - SubT | 0,027561 | 0,012442 | 2,215233 | **0,040116** |
| Metabolites | Ecol - SubT | 0,033471 | 0,012433 | 2,69215 | **0,021298** |
| Nitrogen | Cont - Ecol | -0,08951 | 0,036998 | -2,41942 | **0,023318** |
| Nitrogen | Cont - SubT | -0,15809 | 0,036692 | -4,30844 | **4,93E-05** |
| Nitrogen | Ecol - SubT | -0,06857 | 0,036273 | -1,89047 | 0,058695 |
| Quorum | Cont - Ecol | -0,07063 | 0,041324 | -1,70905 | 0,087442 |
| Quorum | Cont - SubT | 0,129516 | 0,042379 | 3,056126 | **0,003363** |
| Quorum | Ecol - SubT | 0,200141 | 0,042022 | 4,762792 | **5,73E-06** |
